# Case Report: Tricho-hepato-enteric syndrome in an infant presented with colorectal ulceration and severe respiratory superinfection

**DOI:** 10.3389/fimmu.2026.1721204

**Published:** 2026-02-11

**Authors:** Yuta Narishige, Tomohiro Nakano, Yusuke Hoshi, Kenji Sonota, Hiroki Sakurai, Fumihiko Kakuta, Taku Koizumi, Yoji Sasahara, Daiki Abukawa

**Affiliations:** 1 Department of Gastroenterology, Miyagi Children’s Hospital, Sendai, Japan; 2 Department of Pediatrics, Tohoku University Graduate School of Medicine, Sendai, Japan; 3 Department of Intensive Care Medicine, Miyagi Children’s Hospital, Sendai, Japan; 4 Department of Rheumatology and Infectious Disease, Miyagi Children’s Hospital, Sendai, Japan

**Keywords:** combined immunodeficiency, cytomegalovirus, monogenic inflammatory bowel disease, pneumocystis, tricho-hepato-enteric syndrome, very early-onset inflammatory bowel diseases

## Abstract

**Introduction:**

Tricho-hepato-enteric syndrome (THES) is a rare genetic disorder characterized by early-onset intractable diarrhea, intrauterine growth retardation, hair abnormalities, and liver disease during early infancy. THES is often associated with combined immunodeficiency caused by defective interferon-γ production in T cells and hypogammaglobulinemia. However, very few cases of a severe clinical course in infancy have been reported.

**Case description:**

Here, we report the case of a 2-month-old boy who presented with intractable diarrhea, growth retardation, and hair anomaly. Although fasting and central venous nutrition reduced stool frequency, effective weight gain was not achieved. A colonoscopy revealed multiple irregular ulcers without any cytomegalovirus (CMV)-positive cells. Nevertheless, CMV was detected in peripheral blood using a polymerase chain reaction, and the patient was initially treated with ganciclovir. However, this approach was not clinically effective. The second endoscopy revealed new colonic ulcers with mild active inflammation, and treatment with prednisolone was partially effective. The Immunological evaluation revealed no impaired findings, except for low blastogenesis in T cells. However, the patient developed severe progressive respiratory failure caused by superinfection with *Pneumocystis jirovecii* and CMV and died at 6 months of age. Clinical sequencing analysis identified compound heterozygous frameshift variants c.195dupA (p.A66Sfs*3) and c.3426dupA (p.A1143Sfs*4) in *TTC37* (NM_014639.4), confirming the diagnosis of THES.

**Conclusion:**

THES can have a fatal clinical course even during infancy. Detailed immunological and genetic analyses, in addition to endoscopic examination, are crucial for the definitive diagnosis and management of patients with very early-onset inflammatory bowel disease and inborn errors of immunity with systemic features.

## 1 Introduction

Very early-onset inflammatory bowel disease (VEO-IBD) is a type of inflammatory bowel disease (IBD) that manifests in children under the age of 6 years, with approximately 13–20% of cases estimated to have a monogenic etiology (1, 2). Monogenic IBD is frequently associated with inborn errors of immunity, necessitating comprehensive endoscopic mucosal assessment, immunological testing, and genetic analysis in suspected cases. The genetic analysis primarily utilizes targeted sequencing panels, whole-exome sequencing, and whole-genome sequencing (3). Although therapeutic strategies differ across underlying conditions, a key challenge in clinical practice is patient management during the diagnostic process.

Tricho-hepato-enteric syndrome (THES) (NM_014639) is a rare autosomal recessive genetic disorder caused by variants in *TTC37* (OMIM #222470) or *SKIV2L* (OMIM #614602). These variants result in dysfunction of the superkiller complex, leading to aberrant exosome mediated RNA decay and degradation (4). The estimated prevalence of THES is approximately one per million (5). THES is characterized by intractable diarrhea during infancy, growth retardation, hair abnormalities, and liver disease. Histological findings may include villous atrophy of the small intestine and colonic inflammation (5). Although cases presenting with IBD-like phenotypes have been documented in late childhood (6), reports of colonic ulcerations in infancy are rare. Immunological dysfunction in patients with THES is associated with combined immunodeficiency caused by impaired interferon-gamma production by T cells, hypogammaglobulinemia, and attenuated antibody responses (7). However, cases involving infants with severe infections have rarely been reported.

Herein, we report a case of THES in an infant who presented with a VEO-IBD-like phenotype and severe respiratory failure. The patient developed superinfection with *Pneumocystis jirovecii* and cytomegalovirus (CMV) pneumonia at the age of five months, highlighting the complexity of early clinical manifestations of THES.

## 2 Case description

The patient was a male infant born at 38 weeks of gestation via cesarean section because of severe intrauterine growth retardation. He was the second child of dizygotic twins from nonconsanguineous parents, and his brother was healthy. At birth, his body weight was 1,492 g (-4.03 standard deviation [SD]), height was 40.5 cm (-3.26 SD), head circumference was 29.8 cm (-2.5 SD) and Apgar scores were 8 at 1 minute and 8 at 5 minutes. Mild coarctation of the aorta was observed prenatally on ultrasonography; however, no intervention was required. The patient was discharged at 1 month of age, weighing 2,298 g.

**Figure 1:**
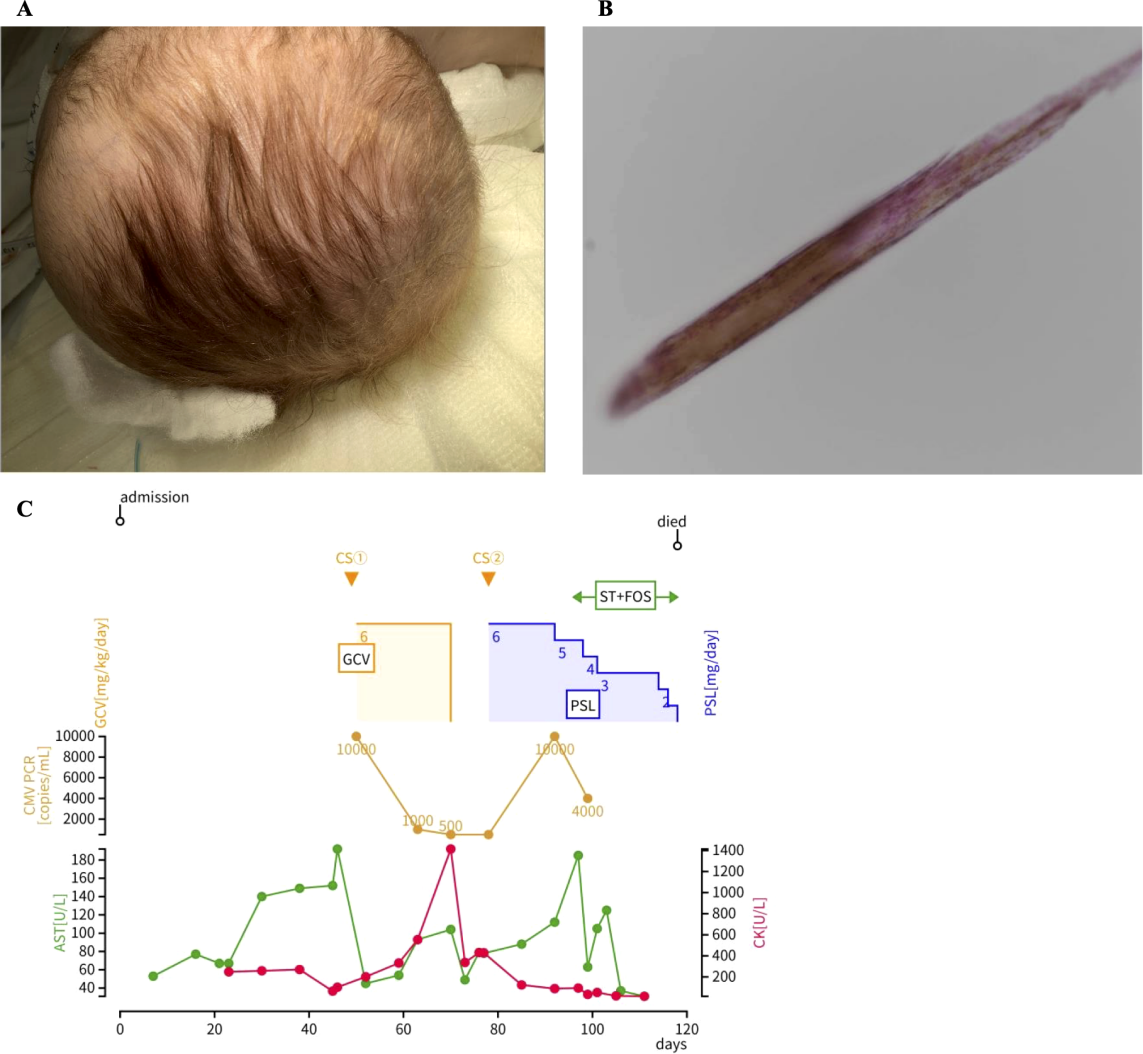
Clinical manifestations and overall clinical course of the patient. **(A)** Hair abnormality at the age of 6 months, **(B)** Microscopic examination of the hair shaft. Light microscopy did not reveal the characteristic nodular deformities (trichorrhexis nodosa) typically associated with THES, and **(C)** summary of the overall clinical course of the patient. CS, colonoscopy; ST, sulfamethoxazole-trimethoprim; FOS, foscarnet; GCV, ganciclovir; PSL, prednisolone; CMV, cytomegalovirus; PCR, polymerase chain reaction; AST, aspartate aminotransferase; CK, creatine kinase.

At 2 months of age, he had persistent diarrhea, > 10 loose stools per day, and vomiting. He had persistent growth failure, with a body weight of 2,403 g (-5.03 SD), a height of 46.7 cm (-5.58 SD), head circumference was 34.0 cm (- 4.12SD). He had light-colored, curly, woolly hair, but trichorrhexis nodosa was not observed (**Figures 1A, B**); no hepatosplenomegaly was observed, and distinctive facial dysmorphism was not prominent. Stool cultures showed normal intestinal flora, and the fecal eosinophil and fat tests yielded negative results. Fecal calprotectin was markedly elevated to 5,565.7 mg/kg. A complete blood count revealed hypochromic microcytic anemia caused by iron deficiency. The liver enzyme levels were within the normal range at the time of admission. Despite the initiation of treatment for refractory diarrhea, including total parenteral nutrition and enteral rest, clinical improvement was limited. Liver enzyme levels gradually increased, although abdominal ultrasonography findings were normal. Overall clinical course was described at **Figure 1C**.

**Figure 2:**
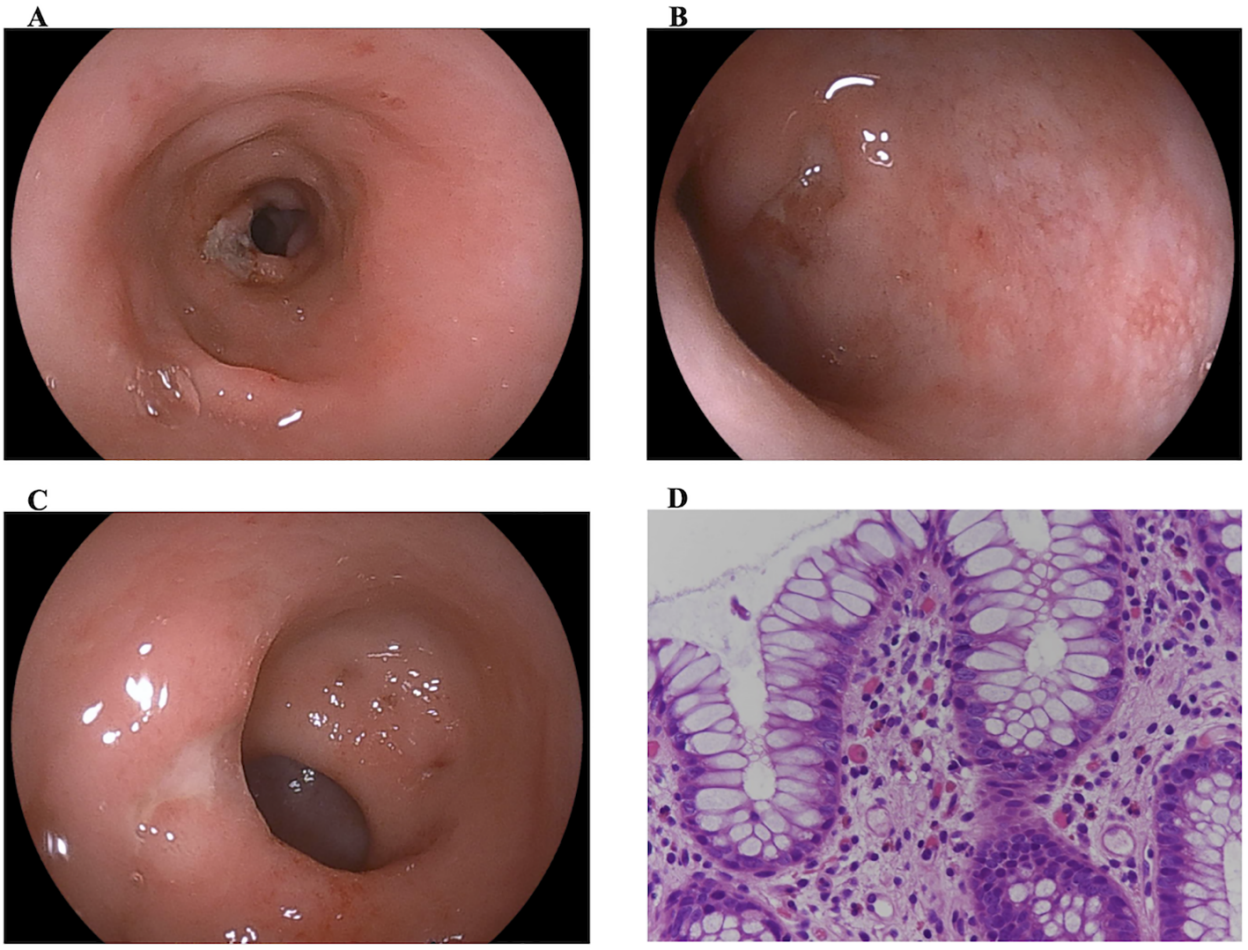
Colonoscopic and histological evaluation of inflammatory bowel disease at onset. **(A–C)** Colonoscopy reveals multiple ulcers in the descending colon and rectum. **(D)** Histopathological examination shows mild inflammation without evidence of apoptosis, and cytomegalovirus is not detected.

At 3 months of age, a colonoscopy revealed multiple ulcers extending from the rectum to descending colon (**Figures 2A–C**). Histopathological analysis revealed mild inflammation without apoptosis (**Figure 2D**). Acquired CMV colitis and monogenic IBD were suspected, and immunological evaluations was initiated. A polymerase chain reaction for CMV on mucosal specimens yielded negative results; however, CMV was detected in the patient’s serum at a concentration of 1 × 10^4^ copies/mL. Neither retinochoroiditis nor cerebral calcification was observed. Given the possibility of acquired CMV colitis, ganciclovir (10 mg/kg/day) was administered for 3 weeks. The treatment was ineffective and was discontinued owing to elevated creatine kinase levels. At 4 months of age, a second colonoscopy revealed new rectal ulcers, prompting reconsideration of the diagnosis. Monogenic IBD, including THES, was suspected. Prednisolone (6 mg/day) was initiated to control intestinal inflammation; however, its effect was limited, and the drug was tapered and discontinued. The patient remained dependent on total parenteral nutrition.

**Figure 3:**
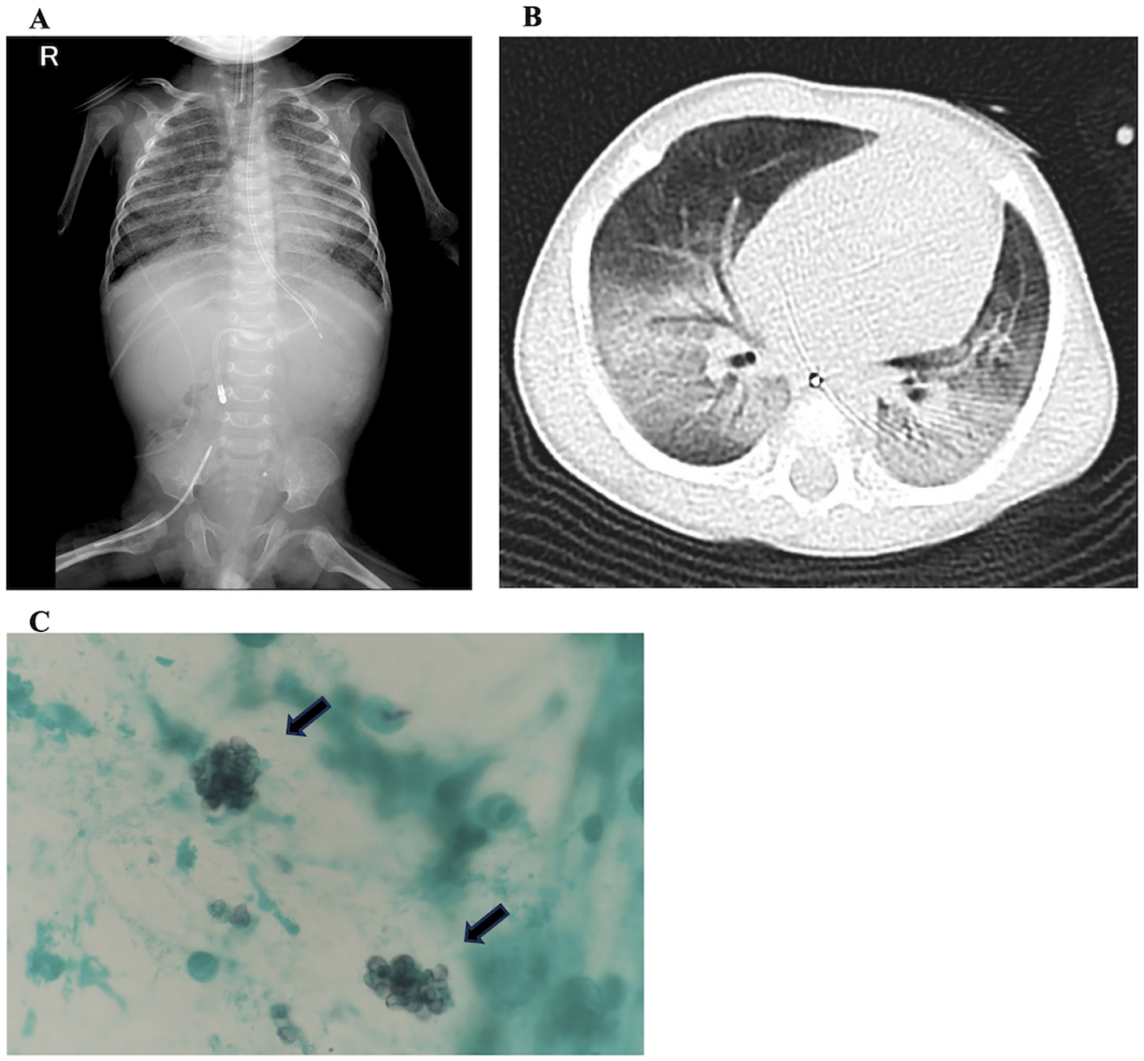
Fatal respiratory distress caused by superinfection with *Pneumocystis jirovecii* and cytomegalovirus. **(A)** Chest radiography and **(B)** computed tomography images show bilateral ground-glass opacities. **(C)** Grocott staining of bronchoalveolar lavage fluid reveals the presence of *P. jirovecii* (arrows).

At 5 months of age, the patient developed respiratory failure. Chest radiography and computed tomography revealed bilateral ground-glass opacities (**Figures 3A, B**). We diagnosed *P. jirovecii* pneumonia based on Grocott staining of the bronchoalveolar lavage fluid obtained during the initial sampling (**Figure 3C**). CMV was detected in the subsequent bronchoalveolar lavage sample using a polymerase chain reaction. Despite intensive treatment, including mechanical ventilation, trimethoprim (15 mg/kg/day), and foscarnet (180 mg/kg/day), respiratory failure progressed, and the patient died at 6 months of age.

The results of immunological evaluation were fully revealed after the patient developed respiratory failure. Normal immunoglobulin levels (IgG, 1,048 mg/dL; IgA, 326 mg/dL; and IgM, 61 mg/dL). Functional assays revealed reduced blastogenesis in response to phytohemagglutinin and concanavalin A stimulation. The copy numbers of the T-cell receptor excision circles (TREC) and kappa-deleting recombination excision circles (KREC), lymphocyte subset analysis, and neutrophil function test results in his peripheral blood were within normal ranges (**Table 1**). An assay for interferon-gamma production in response to stimulation was not performed owing to the limited availability of the patient’s sample.

**Table 1:** Immunological laboratory data.

Parameter	Value	Reference value		Value	Reference value
WBC (/μL)	13,200	4,500–19,000	IFNγ -/IL4 + (%)	1.2	ND
Neutrophils (%)	71.3	ND	IFNγ +/IL4 + (%)	0.4	ND
Monocytes (%)	5.6	ND	IFNγ -/IL4 - (%)	93.1	ND
Lymphocytes (%)	16	ND	IFNγ +/IL4 - (%)	5.3	ND
Total T cells (%)	89	56.6–85.9	T helper/T cytotoxic	1.5	ND
CD3 (%)	59.3	54.3–81.9	memory T helper/% T helper (%)	10.9	15.2–73.5
CD4 (%)	33.5	24.3–49.7	memory T cytotoxic/% T cytotoxic (%)	2.4	5.3–59.8
CD8 (%)	23.7	18.4–49.0	Active HLA-DR+ T cells/% T cells (%)	97.8	83.9–100.0
Total B cells (%)	7.2	2.4–20.4	TCR - αβ/% T cells (%)	97.8	83.9–100.0
Total NK cells (%)	1.7	0.0–23.5	TCR - γδ/% T cells (%)	1.4	0.0–15.3
Serum IgG (mg/dL)	1048	290–960	DNT/% αβ-T cells (%)	0.4	0.0–1.7
IgA (mg/dL)	326	0–33	Switched + memory B cells/% B cells (%)TCR - γδ/% T cells (%)	1.21.4	0.9–24.0
IgM (mg/dL)	61	30–127	IgM + memory B cells/% B cells (%)DNT/% αβ-T cells (%)	5.50.4	0.0–15.7
TREC (copies/mL)	18	>11	IgD + naïve B cells/% B cells (%)Switched + memory B cells/% B cells (%)	90.31.2	56.5–95.3
KREC (copies/mL)	31	>7	IgM + memory B cells/% B cells (%)	5.5	0.0–15.7
Blastogenesis to PHA (SI)	32.9	147.5–1251.3	IgD + naïve B cells/% B cells (%)	90.3	56.5–95.3
ConA (SI)	12.6	38.1–385.5			
Neutrophil phagocytosis (%)	77	40–80			
Neutrophil killing ability (%)	96	70–			

WBC, white blood cells; CD, cluster of differentiation; ND, no data; NK, natural killer; Ig, immunoglobulin; TREC,T-cell receptor excision circle; KREC, kappa-deleting recombination excision circle; PHA, phytohemagglutinin; ConA, concanavilan A; IFN, interferon; IL, interleukin; TCR, T cell receptor; DNT, double negative T cells.

Given that the patient had hair abnormalities, intrauterine growth retardation, growth failure after birth, severe respiratory infections, early-onset intractable diarrhea, and colorectal ulcers, we suspected THES. We performed targeted sequencing analysis for inborn errors of immunity to make a definitive diagnosis using his peripheral blood and identified compound heterozygous frameshift variants c.195dupA (p.A66Sfs*3) and c.3426dupA (p.A1143Sfs*4) in *TTC37* (NM_014639.4).

## 3 Discussion

This case report offers new insights into the diverse clinical spectrum of THES and highlights its potentially severe outcomes. First, severe IBD-like THES may present with significant colonic ulceration even in early childhood. Second, THES can lead to fatal infections during infancy.

Patients with THES typically present with severe refractory diarrhea and faltering growth in early childhood, which may progress to an IBD-like phenotype later in childhood (8). However, it rarely manifests with IBD-like symptoms during early childhood. In the present case, the patient exhibited elevated fecal calprotectin levels and colorectal ulcerations, which led to the diagnosis of VEO-IBD. CMV was not detected in repeated mucosal biopsies. Moreover, the clinical course differed from that of acquired CMV colitis because patients with isolated CMV colitis usually respond well to ganciclovir treatment (9). The colorectal ulceration observed in this case appeared to be associated with THES. While fecal calprotectin levels require careful interpretation in infants, early endoscopic examination and genetic analysis are essential for understanding gastrointestinal symptoms and definitive diagnosis of THES (10). Although a full course of initial antiviral therapy might have prevented the subsequent severe pneumonitis, discontinuation was unavoidable due to adverse events. This highlights the difficulty of managing CMV in THES, where underlying immunodeficiency hinders viral clearance.

The pathogenesis of colorectal ulcerations in patients with THES remains poorly understood. Compared with previously reported cases of IBD-like THES (1, 6, 8), the current patient exhibited the onset of colorectal ulceration at an early age. In this case, compound heterozygous frameshift variants were identified. Although the presence of IBD did not appear to directly worsen the survival prognosis, this patient had a severe phenotype of THES compared with those in other reported cases. These findings highlight the importance of prognostic evaluation in patients with THES, considering IBD presence, disease severity, and detailed genetic assessments. The differential diagnosis for congenital diarrhea and VEO-IBD is broad. We considered conditions such as *DGAT1* deficiency, IPEX syndrome, *SKIV2L* mutations, chronic granulomatous disease (CGD), and *TTC7A* deficiency. Clinically, the patient lacked specific features often associated with these disorders, such as multiple intestinal atresia (*TTC7A* deficiency), severe autoimmunity (IPEX), or abscess formation (CGD). Ultimately, these differentials were definitively excluded based on the targeted sequencing analysis results, which identified pathogenic variants in *TTC37* while revealing no causative mutations in the genes associated with these other disorders.

Patients with THES are predisposed to recurrent infections, which are the primary cause of mortality (11). Although uncommon, fatal infections in infancy have been reported (8). According to the disease classification of inborn errors of immunity, THES is categorized as a syndrome with combined yet variable immunodeficiency (12). We reviewed and summarized the literature on the genotypes and clinical phenotypes of patients with IBD-like THES (**Supplementary Table S1**). Most patients exhibited hypogammaglobulinemia and poor antibody responses after vaccination, necessitating periodic intravenous immunoglobulin replacement therapy. Fatal cases of measles and influenza infections have also been reported. In the present case, intravenous immunoglobulin (IVIG) was not administered because serum IgG levels were preserved. However, given that functional antibody deficiency can occur in THES even with normal immunoglobulin levels, IVIG administration should be considered as an adjunctive therapy during life-threatening infections.

A previous study reported survival probabilities of 92% at 5 years and 81% at 10 years (8). While strict genotype-phenotype correlations remain to be fully established, *TTC37* variants are the most common etiology. Regarding curative intervention, the efficacy of hematopoietic stem cell transplantation (HSCT) appears to be mixed. Although HSCT can normalize immunological defects, it is associated with significant risks and uncertain gastrointestinal outcomes. Reported cases have described procedure-related mortality due to severe infections such as interstitial or adenovirus pneumonia (1, 11). Thus, the indication for HSCT requires a careful assessment of the risk-benefit balance, particularly given the relatively favorable survival with supportive care.

This case was complicated by respiratory superinfection with *P. jirovecii* and CMV, and the patient exhibited a clinical course similar to that of severe combined immunodeficiency (13). However, the patient tested negative on newborn screening tests for TREC and KREC, and no severe functional defects in the T-cell and B-cell lineages were observed, except for low blastogenesis. Based on these results, we concluded that prophylactic antimicrobial treatment was not required at the time.

Although recent developments in newborn screening using TREC and KREC have enabled the diagnosis of inborn errors of immunity before onset of symptoms and appropriate management (14), diagnosing combined immunodeficiency with normal lymphocyte subsets at an early stage remains challenging (15). This case indicates that THES can lead to severe infections despite the absence of obvious immunodeficiency. The case of a patient with normal TREC levels having CMV and *Pneumocystis* superinfection has been reported, similar to this case (16). CMV and *Pneumocystis* infections in immunocompromised individuals are frequently lethal (17, 18). Although genetic testing requires considerable time, early availability of results may help predict severe outcomes. Therefore, we propose that prophylactic antimicrobial treatment should be considered in suspected THES cases until a genetic diagnosis is confirmed, irrespective of the patient’s immunological examination results.

Furthermore, recognizing the triad of refractory diarrhea, IUGR, and hair abnormalities should prompt early comprehensive genetic testing. Rapid diagnosis is vital for considering curative therapies, such as hematopoietic stem cell transplantation, in severe cases.

Clinically, it is crucial to recognize that THES can cause life-threatening infections and present as VEO-IBD symptoms even in early infancy.

## Data Availability

The raw data supporting the conclusions of this article will be made available by the authors, without undue reservation.

## References

[1] Kammermeier J Drury S James CT Dziubak R Ocaka L Elawad M . Targeted gene panel sequencing in children with very early onset inflammatory bowel disease--evaluation and prospective analysis. J Med Genet. (2014) 51:748–55. doi: 10.1136/jmedgenet-2014-102624, PMID: 25194001

[2] Sasahara Y Uchida T Suzuki T Abukawa D . Primary immunodeficiencies associated with early-onset inflammatory bowel disease in Southeast and East Asia. Front Immunol. (2021) 12:786538. doi: 10.3389/fimmu.2021.786538, PMID: 35095863 PMC8792847

[3] Kelsen JR Sullivan KE Rabizadeh S Singh N Snapper S Elkadri A . NASPGHAN position paper on the evaluation and management for patients with very early-onset inflammatory bowel disease (VEO-IBD). J Pediatr Gastroenterol Nutr. (2019) 70:389–403. doi: 10.1097/MPG.0000000000002567, PMID: 32079889 PMC12024488

[4] Fabre A Badens C . Human Mendelian diseases related to abnormalities of the RNA exosome or its cofactors. Intractable Rare Dis Res. (2014) 3:8–11. doi: 10.5582/irdr.3.8, PMID: 25343120 PMC4204543

[5] Fabre A Martinez-Vinson C Goulet O Badens C . Syndromic diarrhea/Tricho-hepato-enteric syndrome. Orphanet J Rare Dis. (2013) 8:5. doi: 10.1186/1750-1172-8-5, PMID: 23302111 PMC3560276

[6] Busoni VB Lemale J Dubern B Frangi F Bourgeois P Orsi M . IBD-like features in syndromic diarrhea/trichohepatoenteric syndrome. J Pediatr Gastroenterol Nutr. (2017) 64:37–41. doi: 10.1097/MPG.0000000000001218, PMID: 28027214

[7] Vély F Barlogis V Marinier E Coste M-E Dubern B Dugelay E . Combined immunodeficiency in patients with trichohepatoenteric syndrome. Front Immunol. (2018) 9:1036. doi: 10.3389/fimmu.2018.01036, PMID: 29868001 PMC5958188

[8] Lee KY Bremner R Hartley J Protheroe S Haller W Johnson T . Long term outcomes in children with trichohepatoenteric syndrome. Am J Med Genet A. (2024) 194:141–9. doi: 10.1002/ajmg.a.63409, PMID: 37753667

[9] Sue PK Salazar-Austin NM McDonald OG Rishi A Cornish TC Arav-Boger R . Cytomegalovirus enterocolitis in immunocompetent young children: A report of two cases and review of the literature. Pediatr Infect Dis J. (2016) 35:573–6. doi: 10.1097/INF.0000000000001080, PMID: 26862673 PMC4829454

[10] Koninckx CR Donat E Benninga MA Broekaert IJ Gottrand F Kolho K-L . The use of fecal calprotectin testing in paediatric disorders: A position paper of the European society for paediatric gastroenterology and nutrition gastroenterology committee. J Pediatr Gastroenterol Nutr. (2021) 72:617–40. doi: 10.1097/MPG.0000000000003046, PMID: 33716293

[11] Girault D Goulet O Le Deist F Brousse N Colomb V Césarini JP . Intractable infant diarrhea associated with phenotypic abnormalities and immunodeficiency. J Pediatr. (1994) 125:36–42. doi: 10.1016/s0022-3476(94)70118-0, PMID: 8021782

[12] Bousfiha A Moundir A Tangye SG Picard C Jeddane L Al-Herz W . The 2022 update of IUIS phenotypical classification for human Inborn Errors of immunity. J Clin Immunol. (2022) 42:1508–20. doi: 10.1007/s10875-022-01352-z, PMID: 36198931

[13] Devonshire AL Makhija M . Approach to primary immunodeficiency. Allergy Asthma Proc. (2019) 40:465–9. doi: 10.2500/aap.2019.40.4273, PMID: 31690396 PMC11275965

[14] de Felipe B Olbrich P Lucenas JM Delgado-Pecellin C Pavon-Delgado A Marquez J . Prospective neonatal screening for severe T- and B-lymphocyte deficiencies in Seville. Pediatr Allergy Immunol. (2016) 27:70–7. doi: 10.1111/pai.12501, PMID: 26498110

[15] Roifman CM Somech R Kavadas F Pires L Nahum A Dalal I . Defining combined immunodeficiency. J Allergy Clin Immunol. (2012) 130:177–83. doi: 10.1016/j.jaci.2012.04.029, PMID: 22664165

[16] Mendez-Echevarria A Gonzalez-Granado LI Allende LM De Felipe B Teresa DR Calvo C . Fatal Pneumocystis jirovecii and Cytomegalovirus infections in an infant with normal TRECs count: Pitfalls of newborn screening for severe combined immunodeficiency. Pediatr Infect Dis J. (2019) 38:157–60. doi: 10.1097/INF.0000000000002058, PMID: 29613974

[17] Vora SB Englund JA . Cytomegalovirus in immunocompromised children. Curr Opin Infect Dis. (2015) 28:323–9. doi: 10.1097/QCO.0000000000000174, PMID: 26098503

[18] Wilson JW Limper AH Grys TE Karre T Wengenack NL Binnicker MJ . Pneumocystis jirovecii testing by real-time polymerase chain reaction and direct examination among immunocompetent and immunosuppressed patient groups and correlation to disease specificity. Diagn Microbiol Infect Dis. (2011) 69:145–52. doi: 10.1016/j.diagmicrobio.2010.10.021, PMID: 21251557 PMC6855182

