# Supplementary material for: Case Report: Tricho-hepato-enteric syndrome in an infant presented with colorectal ulceration and severe respiratory superinfection
Source: Front Immunol. 2026 Feb 11;17:1721204. doi: 10.3389/fimmu.2026.1721204 (PMC12932576; doi:10.3389/fimmu.2026.1721204)
Supplement: Supplementary file 1 [file Table1.docx]

Supplementary Material

**Supplementary Table S1.** Table summarizing the findings of previously published studies.

|  | Present study | Busoni (6) 1 | Busoni (6) 2 | Kammermeier (1) 1 | Kammermeier (1) 2 | Lee (8) 1 | Lee (8) 2 | Lee (8) 3 | Lee (8) 6 | Lee (8) 8 | Lee (8) 11 |
| --- | --- | --- | --- | --- | --- | --- | --- | --- | --- | --- | --- |
| Age at onset | 1 months | 0 months | 1 months | 1 months | 1 months | ND | ND | ND | ND | ND | ND |
| Sex | M | F | F | M | M | F | M | M | F | M | F |
| Endoscopic finding | 4 months: colonic ulcers | 4 months: Pancolitis and rectal sparing | 4 months: Pancolitis 4 years: ulcerative pancolitis | Small bowel, pancolonic disease | Small bowel, pancolonic disease | 7 years: Linear ulcers at the gastro-esophageal junction 13years: Esophagitis, gastritis, focally active colitis | 4 years: Esophageal ulceration, stenosed GOJ distorted pylorus | 7,10 years: Multiple colonic ulcers terminal ileitis | 11 years: Rectal and sigmoid strictures 13 years: colonic ulcers | 11 years, 15 years: Aphthous ulcers in small bowel and pancolitis | 5 years: Duodenal ulceration, anal stenosis and fissuring, multiple colonic ulcers |
| Histology | mild inflammation without apoptosis | 4 months: acute colitis with purulent exudate, cryptitis | 4 months: subtotal villous atrophy without cellular infiltration | Panentric chronic inflammation villus atrophy, crypt epithelial apotosis | Panenteric chronic inflammation, villus blunting epithelial cell irregularity | ND | ND | ND | ND | ND | ND |
| IBD therapy | Steroids | Mesalazine, antibiotics, budesonide, PN | Steroids, mesalazine, antibiotics  anti-TNF-α, PN | Steroids, mesalazine, antibiotics  anti-TNF-α, PN | Steroids, azathioprine, Tacrolimus, sirolimus anti-TNF-α, PN | Steroids, AZA | Steroids, AZA | Steroids, AZA | Steroids, infliximab 5-ASA, AZA | Steroids, infliximab sirolimus | Steroids, 5-ASA azathioprine |
| Gene | *TTC37* | *TTC37* | *TTC37* | *TTC37* | *SKIV2L* | *TTC37* | *TTC37* | *TTC37* | *TTC37* | *TTC37* | *TTC37* |
| Mutation1 (Transcript) | c.195dupA | c.4059delA | c.3808 C>G | c.2018G>A | c.355-2A>C | c.1632+1delG | c.2808G>A | c.2779-2G>A | c.751G>1 | c.2018G>A | c.1135-2A>G |
| Mutation (Protein) | p.A66Sfs*3 | ND | ND | p.Gly673Asp | ND | ND | p.Trp936X | ND | ND | ND | ND |
| Mutation2 (Transcript) | c.3425dupA | c.4575 C>G | homo | c.2808G>A | Homo | homo | Homo | homo | homo | homo | homo |
| Mutation (Protein) | p.A1143Sfs*4 | ND | ND | p.Trp936* | ND | ND |  | ND | ND | ND | ND |
| Infection | CMV pneumonia, PCP led to death | None | none | ND | recurrent bacterial infection | none | Influenza, measles  led to death | ND | ND | ND | ND |
| Immunologic feature | low blastogenesis | low IgG | low IgG | ND | ND | low IgG | None | low IgG | low IgG | low IgG | low IgG |
| Outcome | died at 6 months | 22 years at publish | 7 years at publish | ND | ND | ND | died at 10 years | ND | ND | ND | ND |

IBD, inflammatory bowel disease; ND, no data; GOJ, gastro-oesophageal junction; PN, parenteral nutrition; TNF, tumor necrosis factor; AZA, azathioprine; 5-ASA, 5-aminosalicylic acid; CMV, cytomegalovirus; PCP, pneumocystis pneumonia; Ig, immunoglobulin
